# Understanding the Dynamics of Human Papillomavirus and Diagnostic Discrepancies in Cervical Pathology: A Single Center Experience

**DOI:** 10.3390/diagnostics13243614

**Published:** 2023-12-07

**Authors:** Milena Zamurovic, Ana Tomic, Aleksandra Pikula, Sara Simanic, Aleksandra Knezevic, Marko Jankovic, Milan Lackovic, Elena Djakovic, Marija Rovcanin

**Affiliations:** 1 Clinic for Gynecology and Obstetrics, Narodni Front, Kraljice Natalije 62, 11000 Belgrade, Serbia; 2 Faculty of Medicine, University of Belgrade, Dr Subotica Starijeg 8, 11000 Belgrade, Serbia; 3 Institute of Microbiology and Immunology, Department of Virology, Faculty of Medicine, University of Belgrade, Dr Subotića Starijeg Street, 11000 Belgrade, Serbia; 4 Clinical Hospital Center, Dr Dragiša Mišović, Heroja Milana Tepica 1, 11000 Belgrade, Serbia

**Keywords:** human papillomavirus, screening, colposcopy, cytology, cytological abnormalities, cervical dysplasia, diagnosis, cervical cancer

## Abstract

Cervical cancer (CC) is the most prevalent gynecological malignancy and a leading cause of death among women. It is primarily caused by human papillomavirus (HPV) infection, with 99.7% of cases showing high-risk HPV genotypes. This study sheds light on HPV dynamics as well as the discrepancies of different CC screening modalities results while highlighting factors that may have contributed to such a scenario. Moreover, we underscore the importance of the non-viral etiology of CC as well. We examined the current trends of HPV infection and its effects on cervical health in women treated at a tertiary care center in Belgrade, Serbia. Patients with abnormal colposcopy findings like dysplasia and re-epithelization were more likely to test negative for HPV (*p* < 0.001). Interestingly, women with a positive Pap smear tested HPV negative significantly more often (*p* = 0.041). Finally, HPV-positive individuals were more likely to have CIN I and II histologies (*p* < 0.001), while CIN III occurred equally in women with and without the virus. It may be inferred that inconsistencies in detecting HPV and the presence of cervical lesions may eventually result in adjustments to screening guidelines, as is crucial to adopt a meticulous approach to promote periodical CC screening, as initial samples may test negative for HPV.

## 1. Introduction

Cervical cancer (CC) stands as the prominent gynecological malignancy globally and a significant contributor to female mortality worldwide [1]. It is globally the fourth most common cancer in women [2], ranking second in Serbia after breast cancer [3]. Over 500,000 cases and approximately 300,000 fatalities per year from this illness were reported by the end of the second decade of the twenty-first century [2].

Human papillomavirus (HPV) infection is the leading cause of CC [4]. HPVs are commonly classified into high-risk (16, 18, 31, 33, 34, 35, 39, 45, 51, 52, 56, 58, 59, 66, 68, and 70) and low-risk types based on their association with CC and precursor lesions [5]. Approximately 99.7% of tumors exhibit high-risk HPV (HR HPV) genotypes [6]. HR HPV types disrupt critical cellular processes such as DNA repair and apoptosis, culminating in a gradual progression toward dysplastic changes and eventual malignancy [7]. Considering the gradual evolution of the disease [8] and the fact that not all cases of human papillomavirus (HPV) infection result in the development of cervical cancer (CC) [9], the inquiry regarding other potentially essential cofactors becomes pertinent.

The discovery of the role of HPV in the pathogenesis of CC and subsequent implementation of HPV testing in CC prevention protocols made a significant impact as CC incidence and mortality have declined, especially in developed countries [10], with strategies focusing on the vaccination, screening, and early treatment of dysplasia [11]. The late detection of HPV infection and subsequent alterations in the cervix may be regarded as the primary worldwide cause of CC [1], as ineffective national screening programs significantly correlate with heightened disease incidence [12]. This is particularly noted in low- and middle-income countries that grapple with heightened CC rates due to a lack of organized periodic screening and HPV testing programs [4,10].

Additionally, HPV testing yielded a multitude of other advantages. During the pre-testing period, a rather common disparity between cervical cytology and histology led to diagnostic ambiguity. Cytological errors were commonly identified as the primary cause of these inconsistencies [13,14]. Furthermore, the disparity between cytology and colposcopy findings was influenced by other factors, such as age and HPV genotypes [15]. The presence of cytohistological discrepancies in women with high-risk cervical lesions is a matter of utmost significance, as it could lead to a delay in treatment if less aggressive management is pursued. The HPV detection test is regarded as a crucial diagnostic approach to address these inconsistencies [13].

Due to its sensitivity in detecting precursors with risk stratification based on HPV genotypes, global guidelines implemented primary HPV testing as the principal method of screening [16]. However, certain discrepancies reappeared. The possibility of HPV false-negative high-grade cervical lesions that might go undetected with HPV primary screening justified reservation [17]. Primary HPV testing may fail to identify certain cases, but these cases can be discovered with subsequent PAP smear tests. A screening policy where women who tested positive for HPV were further evaluated using the PAP smear assessment and biopsy led to an almost threefold increase in the diagnosis of high-grade cervical pathologies [18]. It is crucial to examine any discrepancies in the diagnosis of cervical pathologies as well as contributing factors in order to prevent unnecessary and extensive examination as well as the underdiagnosis of high-grade cervical lesions [15].

For resource-constrained countries, the alternative practice of HPV/Pap co-testing, which involves the simultaneous administration of an HPV DNA test and a Pap test, is established with the purpose of early detection of cervical lesions [19,20]. Serbia, positioned as a middle-income nation [21], necessitates evidence-based screening protocols in alignment with prevailing HPV infection trends, as HPV genotypes and clinical manifestations show evident geographical diversity [22].

Herein, we inquired into the current trends of HPV infection and cervical disease manifestations in Serbian women diagnosed with HPV, complementing the findings with experiences with unconventional discoveries and ultimately in relation to the overall context of current global guidelines for HPV screening for CC prevention.

## 2. Materials and Methods

### 2.1. Study Design

To obtain data on HPV prevalence, a cross-sectional study on a population of adult Serbian females was carried out. The frequency of HPV genotypes and their association with findings obtained by colposcopy, histopathology, and cytology were also investigated. Additional data for the period of 2019–2022 were obtained from a comprehensive database that was established in 2019 to document and store information pertaining to the total count of HPV-positive findings observed annually as well as the specific types of viruses identified within each respective year.

### 2.2. Participants

The cohort consisted of 310 female patients who were diagnosed at outpatient primary care centers and referred to the “Narodni front” Obstetrics and Gynecology Clinic in Belgrade, Serbia, between January and December 2022. All the patients with cytological or colposcopic abnormalities who were previously identified during standard gynecological checks in the primary care evaluation centers were subsequently referred to our clinic for further evaluation and treatment. After re-examinations in our clinic, only validated pathological findings were followed by HPV genotyping. Data on vaccination against HPV were obtained using a general questionnaire created for the purpose of this study. The exclusion criteria were as follows: (1) follow-up examinations; (2) colposcopy referral for other gynecological conditions such as opportune screening; (3) small, regular colposcopic and Pap findings; and (4) colposcopic changes that could not be associated with the presence of HPV infection. Furthermore, this study did not include patients who had first received a diagnosis of abnormality by colposcopic examination and/or pathological Pap test in primary health care but later had this diagnosis disproven via examination in our clinic.

### 2.3. Data and Sample Collection

#### 2.3.1. Questionnaire Data Collection

A general questionnaire was used to gather information on the patient sociodemographic characteristics (age, education, employment, marital status, socioeconomic status), habits, and gynecologic and obstetric conditions.

#### 2.3.2. Colposcopy, Cytology, and Histopathology Analyses

The Pap smear test was performed using the conventional method. The sample was evaluated for cytologic evidence of neoplastic changes that were reported using the Bethesda system for the classification of cervical cytology [23]. The specimen was subsequently classified into one of the five groups. The Pap smear was followed by a routine colposcopy. Only smears exhibiting abnormal cytological findings were subsequently subjected to HPV detection by PCR. In the case of an abnormal Pap (aPap) smear result (higher grade of pathological lesions than ASCUS), a colposcopy with a punch biopsy was performed for further histopathological evaluation. A diagnosis of cervical intraepithelial neoplasia (CIN) was based primarily on the presence of nuclear atypia and loss of normal squamous maturation. CIN lesions were graded based on the proportion of cervical epithelium exhibiting dysplastic cells and accordingly classified as low-grade (CIN 1) and high-grade (CIN 2 and 3) [24].

#### 2.3.3. HPV Sampling, Detection, Typing and Data Storage

Sample collection for HPV genotype testing was carried out at the Obstetrics and Gynecology Clinic “Narodni front” in Belgrade, Serbia, with molecular analysis done at the Institute of Microbiology and Immunology of the Faculty of Medicine, University of Belgrade.

Using cervical cytobrush techniques (Cervex-Brush^®^ Combi, Rovers, The Netherlands), endocervical swabs and/or swabs from visible lesions in the ectocervix were collected to determine the presence of HPV infection. To keep the sample’s humidity constant, the swabs were placed in tubes with 1 mL of transport medium. “Squeezing of the cytobrush” in a specialized medium (liquid-based cytology samples, CytoScreen, preserve, Cyt) was required for additional processing in order to conduct viral testing. Following that, the samples were centrifuged at 2000–3000 rpm. Total DNA was isolated from the resultant sediment using a commercial DNA extraction kit (QIAamp DNA Mini Kit, QIAGEN Inc., Germantown, MD, USA) in accordance with the manufacturer’s instructions. Two sets of primers, MY09/MY11 and GP1/GP2, were used for the amplification of the HPV *L1* and *E1* genes. The QiagenTaq PCR Master Mix (QIAGEN Inc., Germantown, MD, USA), 1 mol of each HPV primer, 0.5 mol of each β-globin primer, and 5 μL of extracted DNA were added to a 25 μL volume reaction mix to conduct the PCR [25,26]. Initial denaturation at 95 °C for 5 min was followed by 40 cycles of 30 s at 94 °C, 30 s at 58 °C, 1 min at 72 °C, and a final elongation of 20 min at 72 °C in the PCR technique for the detection of the *L1* gene [25]. The technique for detecting the *E1* gene included a 5 min initial denaturation at 85 °C, followed by 40 cycles of 1 min at 94 °C, 1 min at 50 °C, 90 s at 72 °C, and a final elongation of 10 min at 72 °C [26]. Samples were considered positive for HPV DNA if distinct 450 bp bands for the *L1* and *E1* genes were found using agarose gel electrophoresis and ethidium bromide staining. Any specimen that tested negative for both the HPV amplicon and the β-globin amplicon was deemed inadequate.

Direct DNA sequencing was used for HPV genotyping. According to the manufacturer’s instructions, HPV-positive samples were isolated using the QIAGEN MinElute PCR Purification Kit (QIAGEN Inc.). Using the same set of primers, the Big Dye Terminator 3.1. Cycle Sequencing Kit (PE Applied Biosystems, Waltham, MA, USA) was used to sequence the purified PCR results. On the ABI Prism 310 Genetic Analyzer, sequencing reactions were analyzed. Software called Sequence Analysis 5.1 was used to analyze the retrieved nucleotide sequences. Using the BLAST program v. 2.15.0+ (http://www.ncbi.nlm.nih.gov/BLAST/; accessed on 20 January 2023), HPV genotypes were identified by comparison with HPV reference strains and reported viral sequences available in the GenBank database (release 258.0). If the nucleotide sequence matched a known HPV genotype more than 95% of the time, the HPV type was determined.

Upon the completion of the microbiological analysis of the samples, the obtained findings were subsequently forwarded to the Obstetrics and Gynecology Clinic “Narodni front” Belgrade, Serbia, where a comprehensive database was established in 2019 to document and store information pertaining to the total count of positive findings observed annually as well as the specific types of viruses identified within each respective year.

### 2.4. Ethical Consideration

This study was implemented in accordance with the International Code of Medical Ethics of the World Medical Association (Declaration of Helsinki), and written informed consent was obtained from the participants after the nature and objectives of this study were fully explained to them. This study was approved by the institution’s Ethical Committee (decision number 22008-2023-009350; date of approval: 1 December 2021).

### 2.5. Data Analysis

Descriptive statistical methods were used to characterize the patient’s sociodemographic and clinical data, presented as counts and percentages. The numeric variables were not normally distributed and are presented as medians with an interquartile range (IQR), while categorical variables were described using absolute and relative frequencies, *n* (%). The ranked data, which were derived from the counts of each test result category, are presented as numbers and percentages. We conducted comparisons between HPV- positive and negative groups using Pearson’s chi-squared tests for frequencies. Statistical analysis was performed using IBM SPSS Statistics for Windows, version 23.0 (IBM Corp., Armonk, NY, USA). The significance level was set at α = 0.05. All 310 participants were included in this analysis.

Age was expressed as the median and IQR and as a categorical variable with two age groups: 18–29 and 30–65. In order to present the differences in the findings with emphasis on the patient’s age suitable for screening, the test subjects were divided into younger than 30 years old and above the given age. The division is carried out in relation to findings that the peak for primary HPV infections is for women who are younger than 30 years old [19]. The grades of neoplastic lesions were treated as proportions and categorical variables. HPV presence was treated as a categorical variable, forming two main groups of interest, HPV-positive and HPV-negative samples, which were then stratified by the Pap smear test result, the colposcopy results of pathological lesions, and the prevalence of histopathology groups with the following normal findings: low-grade squamous intraepithelial lesion (LSIL)—CIN; high-grade squamous intraepithelial lesion (HSIL)—CIN II and CIN III lesions. The corresponding prevalence was calculated for each result.

## 3. Results

### 3.1. Distribution of Results According to Age

The median age of our subjects was 38 with an interquartile range of 30–44 years, where the oldest subject was 69 years old and the youngest was 19. The median age of menarche onset was 13, where the earliest period of menarche was 10 years old and the latest was 19 years old.

For the reasons previously described in the methodology and for the sake of an easier examination and presentation of the results, the subjects were divided into two age groups: 18–29 (*n* = 76) and 30–65 years (*n* = 234). There was no statistically significant difference between these two age groups regarding the Pap test results (χ^2^ = 0.989, *p* = 0.320), histopathological findings (χ^2^ = 3.382, *p* = 0.336), and HPV prevalence frequency of detection of the virus itself (χ^2^ = 3.048, *p* = 0.081). A statistically significant discrepancy was solely seen in the distribution of distinct colposcopic findings. (χ^2^ = 12.046, *p* = 0.002). Dysplasia and re-epithelialization were significantly more prevalent in the older group, as seen in Table 1. Notably, in both age groups, dysplasia was the dominant colposcopy finding.

### 3.2. HPV Prevalence Based on Participant Sociodemographic and Clinical Features

Among the total of 310 participants, 205 (66.1%) individuals tested negative for human papillomavirus (HPV), while the remaining 105 (33.9%) tested positive for HPV. HPV positivity did not differ significantly regarding subject marital status, education level, active smoking, alcohol consumption, employment, or socioeconomic status. Significantly fewer previous births were reported in the HPV-positive group (χ^2^ = 4.845, *p* = 0.028). Abortions were not significantly related to HPV status (χ^2^ = 0.474, *p* = 0.491) (Table 2). 

### 3.3. Overall HPV Prevalence Based on the Pap Smear, Colposcopy, and Biopsy Results

The analysis shown in Table 3 revealed a statistically significant difference in the Pap test groups (χ^2^ = 4.158, *p* = 0.041), colposcopy findings (χ^2^ = 22.425, *p* < 0.001), as well as in the biopsy findings (χ^2^ = 7.936, *p* = 0.019), depending on whether the HPV virus was detected in the sample.

As observed in relation to pathological findings, patients with ASCUS/ASCH smears were HPV negative substantially more often (61.4%). A higher proportion of patients who demonstrated re-epithelialization and precancerous (dysplastic) lesions during colposcopy were also found to be HPV-negative (69.4% and 52.3%, respectively).

Normal HP findings were HPV negative in 163 (98.8%) cases. All observed pathological HP findings were substantially more frequent in the HPV-positive group. LSIL (CIN I) was a dominant HP finding among HPV-positive samples as well as among HPV-negative samples. When considering only the CIN III findings, both HPV-negative and HPV-positive samples were represented in nearly identical proportions, accounting for 48.5% and 51.5%, respectively.

### 3.4. HR HPV Based on the Pap Smear, Colposcopy, and Biopsy Results

As seen in Table 4, the oncogenic potential of the detected HPV genotype did not affect the frequency of certain findings on the Pap test, colposcopy, or pathological biopsy, nor were the abnormal ones significantly more common. HR HPV strains were not statistically more frequent in any age group. Notably, only two HPV-positive samples had normal HP findings and were infected by LR HPV types.

### 3.5. HPV Genotype Trends in Serbia in Correlation to Sample HPV Types

**Figure 1:**
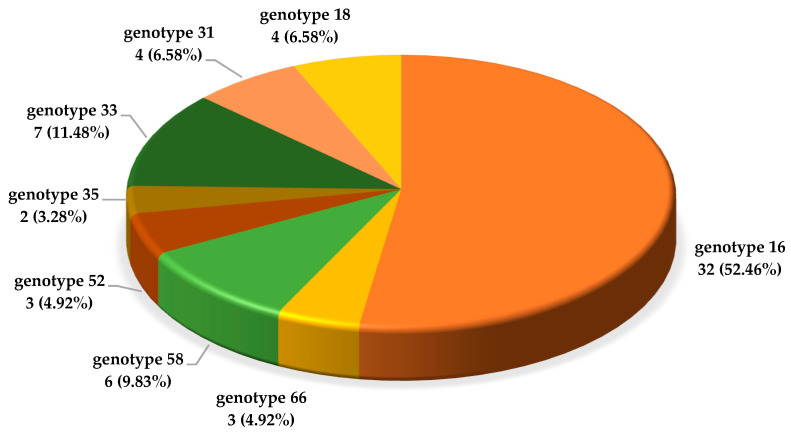
Frequency distribution of high-risk (HR) HPV genotypes in our sample.

Of the 310 samples, HPV was detected in 105 (33.3%). A single HR HPV type was present in 62 (59%) samples. Genotype 16 was a dominant viral type and was detected in 32 cases (52.5%), while the other genotypes were relatively evenly distributed in the remaining cases, including types 33 (11.5%), 58 (9.8%), and 31 (6.56%) (Figure 1).

**Figure 2:**
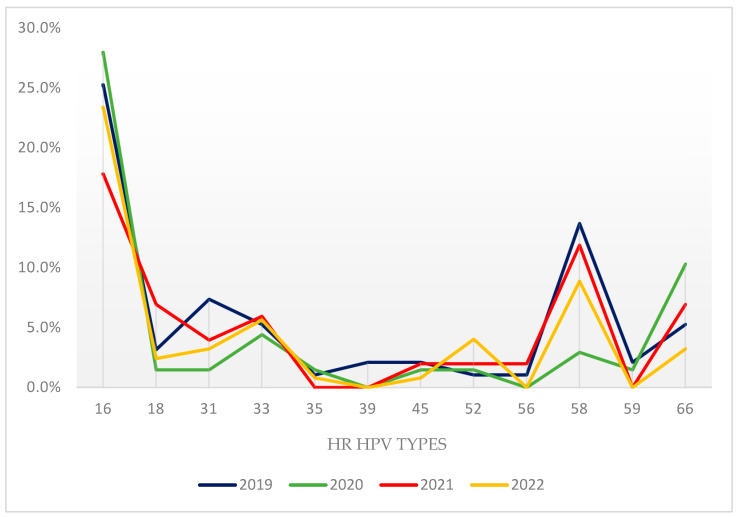
The percentage representation of distinct high-risk (HR) HPV types out of all detected HPV genotypes in cervical lesions during the 2019–2022 period at “Narodni front” Obstetrics and Gynecology Clinic in Belgrade, Republic of Serbia. Note the overall prevailing pervasiveness of types 16, 58, 33, 31, and 66.

Figure 2 illustrates the incidence of different HR HPV genotypes based on all detected HPV types in samples from 2019 to 2022. Considering their importance in the pathogenesis of primarily gynecological cancers, we can distinguish particularly frequent HR HPV strains. The prevalence of types 16, 58, 33, 31, and 66 exhibited a peak throughout the follow-up period. In 2022, the most frequently identified human papillomavirus (HPV) types were 16 (23.4%) and 58 (8.9%), followed by types 33 (5.6%), 31 (3.2%), and 66 (3.2%).

## 4. Discussion

This study addressed the molecular epidemiology of HPV infection in our study population and investigated the correlation between HPV infection and selected clinical parameters. The findings of our study were subjected to a thorough review in accordance with the prevailing international and domestic guidelines for CC screening. Regarding HR HPV genotypes, type 16 was the most prevalent among high-risk genotypes (Figure 1), as evidenced in other research across different populations and nationalities as well [22,27]. This finding is of great importance as the HPV 16 genotype is directly responsible for the recent rise in the incidence of squamous cell carcinoma and adenocarcinoma of the cervix, particularly in younger age groups [28].

The aforementioned statement holds true for HPV types 58, 31, and 33 as well (Figure 1). These types have been found to exhibit higher prevalence rates among females residing in developing countries, in contrast to those in high-income regions [29]. The significance of this matter lies in the fact that these particular types serve as indicators for an increased likelihood of experiencing higher rates of morbidity in relation to cervical CIN II and CIN III lesions [30]. On the other hand, serotype 18 prevalence was less than 7% in our study group. While studies conducted at the beginning of the 21st century reported that HPV type 18 was identified as the second most prevalent serotype [27,31], the majority of studies carried out within the past five years have consistently reported a low occurrence rate of type 18 [22,32,33].

In our study, the presence of an abnormal clinical result was not strictly associated with HR HPV types. This is at odds with evidence that women who have been infected with high-risk strains of HPV are predicted to have a higher probability of experiencing severe dysplastic alterations [27]. Our findings supported previous reports showing more than half of detected HPV types in screened populations were cancer-causing [34,35,36], but there was no evidence linking them to pathological findings. This deviates from the findings of earlier studies with similar cohort sizes, which found a correlation between cervical lesions and HPV, namely its genetic material and high viral load [37]. This might be highly relevant, but because our study was cross-sectional, it should be interpreted with caution because a follow-up period could deliver different results.  

Nevertheless, HPV typing will always have the foremost epidemiological significance, as nationally dominant types (particularly high-risk ones) may influence vaccine HPV genotype profiles [38].

The results of our study suggest that there is no statistically significant variation in the occurrence of HPV-positive samples or abnormal biopsy outcomes based on the age of the participants. These results contradict literature-based expectations that the peak infection incidence occurs in individuals aged 25 to 35 years [39,40,41], with approximately 80% of cases being transient and resolving spontaneously within a year without the need for treatment [42], which leads to a negative HPV result [43]. Moreover, women over the age of 45 are a risk group for inconsistencies in cervical cytohistology results, i.e., aPap smears that are negative for HSIL lesions from a follow-up biopsy [15]. As the patient age in our study was skewed towards an older population, this correlates with overall lower HPV positivity.

Interestingly, the frequency of aPap smears was significantly higher in HPV-negative samples compared to positive ones. Several factors have been identified as causes for misinterpreting cytology findings in HR HPV-positive samples that exhibit negative for intraepithelial lesion or malignancy (NILM) on a Pap smear but have an obscured HSIL cervical lesion. Essentially, the absence of diagnostic cells, unsatisfactory samples, and interpretation variances that are particularly influenced by marked obscuring inflammation lead to diagnostic inconsistencies when examining cytology samples that require adequate rescreening [14].

Furthermore, precancerous CIN III lesions were also more often HPV negative than would be expected. It has been previously observed that certain individuals diagnosed with CC who had previously received positive outcomes from Pap tests, colposcopies, and biopsies also exhibited negative HPV results [44]. Cases of aPap HPV-negative samples that present with CIN III lesions are rare overall, their significance lies in the fact that they represent instances that would be overlooked by primary HPV testing but would be detected by HPV/cytology co-testing with subsequent biopsy [17].

CIN II and CIN III may have the virus at lesser quantities, below PCR detection strength, which impinges on detection rates, or the virus has been already eliminated from the majority/all of the cells, but the visible pathological changes persist. Furthermore, sampling methods might have been the reason behind lessened detectability, as sampling might have targeted lesions specifically, or a routine cervical swab has been performed. Finally, the conspicuous lack of HPV might stem from the PCR tests used [25,26], which are not validated for in vitro diagnostics (IVD). We would be remiss, however, not to mention that, at this time, there are no state-regulated IVDs for the detection of HPV in Serbia up to this point.

Given that almost half of the abnormal clinical outcomes were reported as HPV negative, it follows that although HPV infection is the primary known CC cause, there may be other variables that could aid neoplastic transformation [45], such as multiple or high-risk sexual partners, cigarette consumption, diet, inadequate socioeconomic factors, usage of oral contraception, immunosuppression (such as in HIV infection), or positive history for sexually transmitted diseases or other genital cancers [1,4]. Apart from known associated risk factors, certain bacteria could aid neoplastic transformation. It was shown that *Chlamydia trachomatis* infection was significantly associated with the development of cervical cancer [46]. While the precise mechanisms by which cervical cancer develops due to factors other than HPV are not yet fully understood, it is widely recognized that an imbalance in the vaginal microbiome plays a significant role in its occurrence and progression. [47,48].

As expected, normal biopsy and colposcopy findings were dominant within HPV-negative samples, while abnormalities were mostly observed within the HPV-positive group, which accords with other reports [49]. The proportions of HPV positives and HPV negatives in the colposcopy-registered dysplasia and biopsy CIN III sample pools were nearly identical. The vast majority of studies indicate that nearly all CIN III cases have detectable HPV [50,51], while certain authors have documented instances of CC and CIN III lesions that are HPV negative [52]. Moreover, distinct HR HPV types can impact the results of screening causing diagnostic and management doubts. Cervical infections caused by high-risk HPV types other than types 16 and 18 tend to display certain discrepancies, where abnormal cytology is most likely accompanied by LSIL (CIN I) lesions, with an even more unlikely scenario for them to be presented with a normal Pap smear with insidious HSIL (CIN II and CIN III) lesion [15].

Depending on histopathology, HPV prevalence in CC may vary. While squamous cell carcinomas are almost 100% HPV positive, adenocarcinomas are around 83%, with certain, although rather rare, subtypes being positive in 0–30% of cases [53]. Cervical adenocarcinoma is considered more aggressive, highly recurrent, with more frequent distal metastases, and associated with worse treatment outcomes [39,54]. All of the above might hint that, alongside HPV, there are many other variables that influence the development of neoplasia [45]. Along with different courses based on histopathology, national screening policies must be carefully developed, as no CC case should be left undetected by regular screening.

Over half of reported aPap smears and precancerous colposcopy results were HPV-negative. If these results were to be consistent on a national scale, Serbian primary screening should likely include both HPV and Pap smear testing from a single sample (referred to as co-testing, as presented in the literature), as it is approved as one of the screening alternatives by the accepted guidelines [19]. Moreover, co-testing presents certain advantages over isolated HPV testing. Studies showed that both HPV testing and cytology increased the sensitivity and specificity of CC screening while lowering false-positive cases [55], with the potential to lower the rates of diagnostic inconsistencies [17]. This, in turn, ensures that most CCs will be detected during subsequent diagnosis [56,57]. The integration of cytology and HPV genotyping detection offers a more efficient approach to assessing the morbidity risks associated with cervical CIN II and III lesions [30]. Additionally, study findings indicate that the utilization of co-testing has the capacity to yield enhanced clinical and economic outcomes in comparison to the exclusive use of HPV primary testing [58]

The absence of age-based differences in HPV infection and concomitant cervical lesions brings up the topic of the optimal age to initiate regular co-testing. As HSIL (CIN II and CIN III) occurred in our younger age group and were more often infected with HR HPV types, we concord with the guidelines suggesting screening in their early twenties rather than at the age of 30 [16,19], even though it may be the most cost-effective choice for an implemented screening policy [58]. Even though CIN I and CIN II lesions regress in around 50–60% of cases [59], they should not be missed as they require follow-up over time, reminding us to diligently promote the worldwide recognized importance of consistently advocating for prompt introduction and regularly repeated screening at intervals of three to five years [19].

There are certain limitations to the current study that should be mentioned. The cohort size was small, and the findings differ from those recorded in the literature, which demands testing in a bigger cohort in future investigations. Furthermore, a small cohort may be biased towards a lower HPV prevalence than expected. As this study was cross-sectional, a follow-up period could deliver different findings, so the current results should be adopted with caution. Hence, more longitudinal investigations are required in order to enhance understanding and provide detailed elucidation of the stated results. Given that individuals who have cervical abnormalities identified during colposcopy or receive an abnormal Pap test result during primary care evaluation are subsequently referred to our clinic or tertiary care facility for further evaluation and treatment, it is crucial to emphasize the presence of referral bias in the selection of patients for inclusion in this study. Moreover, the exclusion of individuals with small and regular colposcopy and Pap findings may accidentally result in the removal of instances with lower-grade abnormalities that are nevertheless relevant to the study inquiry. Hence, it is possible that the research participants may not provide an adequate representation of HPV prevalence in the broader community, thus necessitating the need for multicenter studies to assess the validity of these findings on the national level.

## 5. Conclusions

Human papillomavirus infection remains the primary causative agent of CC. However, the need for careful consideration of other contributing factors is essential, as our findings reveal that nearly half of the significantly abnormal clinical outcomes were identified as HPV negative. This indicates that primary national, large-scale longitudinal studies with follow-up periods are necessary for an exact representation of HPV prevalence in pre-CC cervical pathologies. Such discrepancies in HPV detection and the existence of cervical lesions could later lead to the adaptation of screening recommendations on a national level in order to institute appropriate prevention strategies, primarily insisting on both HPV testing and Pap smear testing.

Finally, an efficient, constant, and diligent approach to the promotion of regular screening—even for women with initially negative samples—is essential in advancing good health practices in combating HPV and CC.

## Figures and Tables

**Table 1:** Sample results in relation to different age groups.

		Age Groups						
		19–29 Years		30 Years and above		Total		
		*N* %		*N* %		*N* %		*p*-Value *
HPV detection	HPV (−)	44	(21.5%)	161	(78.5%)	205	(100.0%)	0.081
	HPV (+)	32	(30.5%)	73	(69.5%)	105	(100.0%)	
Pap smear test result	NILM	33	(27.3%)	88	(72.7%)	121	(100.0%)	0.320
	aPap	41	(22.3%)	143	(77.7%)	184	(100.0%)	
Colposcopy	Normal findings	17	(20.2%)	67	(79.8%)	84	(100.0%)	0.002 *
	Re-epithelialization	15	(15.3%)	83	(84.7%)	98	(100.0%)	
	Dysplasia	44	(34.4%)	84	(65.6%)	128	(100.0%)	
Histopathology finding	Normal Finding	39	(23.6%)	126	(76.4%)	165	(100.0%)	0.336
	LSIL (CIN I)	25	(25.5%)	73	(74.5%)	98	(100.0%)	
	HSIL (CIN II)	6	(42.9%)	8	(57.1%)	14	(100.0%)	
	HSIL (CIN III)	6	(18.2%)	27	(81.8%)	33	(100.0%)	

HPV(−)—HPV negative; HPV(+)—HPV positive; aPap—abnormal Pap smears; NILM—negative for intraepithelial lesion or malignancy; LSIL—low-grade squamous intraepithelial lesion; HSIL—high-grade squamous intraepithelial lesion; CIN—Cervical Intraepithelial Neoplasia; * *p*-value based on Pearson’s chi-squared test; * significant at <0.05.

**Table 2:** Sociodemographic and clinical features.

		Virus Detection				
		HPV-Negative		HPV-Positive		
		N	(%)	N	(%)	*p*-Value *
Marital status	Married	152	(66.1%)	78	(33.9%)	0.979
	Extramarital Union/Single	53	(66.3%)	27	(33.7%)	
Education	Highschool/Bachelor	98	(71.0%)	40	(29.0%)	0.104
	University/Master/PhD	107	(62.2%)	65	(37.8%)	
Employment	No	22	(71.0%)	9	(29.0%)	0.548
	Yes	183	(65.6%)	96	(34.4%)	
SES	Good	156	(66.4%)	79	(33.6%)	0.915
	Average/Bad	49	(65.3%)	26	(34.7%)	
Smoking	No	113	(64.6%)	62	(35.4%)	0.509
	Yes	92	(68.1%)	43	(31.9%)	
Alcohol consumption	No	159	(65.4%)	84	(34.6%)	0.621
	Yes	46	(68.7%)	21	(31.3%)	
Previous deliveries	No	42	(57.5%)	31	(42.5%)	0.028 *
	Yes	70	(73.7%)	25	(26.3%)	
Previous miscarriages	No	70	(65.4%)	37	(34.6%)	0.491
	Yes	41	(70.7%)	17	(29.3%)	

SES, socioeconomic status; * *p*-value based on Pearson’s chi-squared test; * significant at <0.05.

**Table 3:** HPV prevalence on Pap smear, colposcopy, and biopsy.

		Virus Detection						
		HPV-Negative		HPV-Positive		Total		
		N (%)		N (%)		N (%)		*p*-Value *
Pap smear test result	NILM	88	(72.7%)	33	(27.3%)	121	(100.0%)	0.041 *
	aPap	113	(61.4%)	71	(38.6%)	184	(100.0%)	
Colposcopy	Normal findings	70	(83.3%)	14	(16.7%)	84	(100.0%)	<0.001 *
	Re-epithelialization	68	(69.4%)	30	(30.6%)	98	(100.0%)	
	Dysplasia	67	(52.3%)	61	(47.7%)	128	(100.0%)	
Histopathology finding	LSIL (CIN I)	23	(23.5%)	75	(76.5%)	98	(100.0%)	0.019 *
	HSIL (CIN II)	3	(21.4%)	11	(78.6%)	14	(100.0%)	
	HSIL (CIN III)	16	(48.5%)	17	(51.5%)	33	(100.0%)	

aPap—abnormal Pap smears; NILM—negative for intraepithelial lesion or malignancy; LSIL—low-grade squamous intraepithelial lesion; HSIL—high-grade squamous intraepithelial lesion; CIN—cervical intraepithelial neoplasia; * *p*-value based on Pearson’s chi-squared test; * significant at <0.05.

**Table 4:** Oncogenic HPV types prevalence based on age, Pap smear, colposcopy, and biopsy.

		HPV Serotypes						
		Low Risk		High Risk		Total		
		N (%)		N (%)		N (%)		*p*-Value *
Age groups	19–29	14	(43.8%)	18	(56.3%)	32	(100.0%)	0.700
	30 and above	29	(39.7%)	44	(60.3%)	73	(100.0%)	
Pap smear test	NILM	16	(48.5%)	17	(51.5%)	33	(100.0%)	0.314
	aPap	27	(38.0%)	44	(62.0%)	71	(100.0%)	
Colposcopy	Normal findings	6	(42.9%)	8	(57.1%)	14	(100.0%)	0.852
	Re-epithelialization	11	(36.7%)	19	(63.3%)	30	(100.0%)	
	Dysplasia	26	(42.6%)	35	(57.4%)	61	(100.0%)	
Histopathology finding	LSIL (CIN I)	33	(44.0%)	42	(56.0%)	75	(100.0%)	0.304
	HSIL (CIN II)	3	(27.3%)	8	(72.7%)	11	(100.0%)	
	HSIL (CIN III)	5	(29.4%)	12	(70.6%)	17	(100.0%)	

aPap—abnormal Pap smears; NILM—negative for intraepithelial lesion or malignancy; LSIL—low-grade squamous intraepithelial lesion; HSIL—high-grade squamous intraepithelial lesion; CIN—cervical intraepithelial neoplasia; * *p*-value based on Pearson’s chi-squared test; * significant at <0.05.

## Data Availability

The data presented in this study are available on request from the corresponding author.
